# From Poppers to Peril: Recreational Nitrite-Induced Methemoglobinemia

**DOI:** 10.7759/cureus.67435

**Published:** 2024-08-21

**Authors:** Wasef Alkhateeb, Gaurav Luthra, Lanah Almatroud, Alyaa Saleh, Elise Landa

**Affiliations:** 1 Internal Medicine, Central Michigan University College of Medicine, Saginaw, USA; 2 College of Human Medicine, Michigan State University, East Lansing, USA; 3 Critical Care Medicine, Ascension Providence Hospital, Southfield, USA

**Keywords:** inhalant abuse, green urine, substance abuse, methylene blue, poppers, methemoglobinemia, inhaled nitrites

## Abstract

Inhaled amyl nitrites, once used to alleviate chest pain, are more commonly now being used for their euphoric effects. The recreational use of inhaled nitrites can lead to methemoglobinemia, a life-threatening condition that hinders the utilization of oxygen in the body. In 2021, 2.2 million people aged 12 or older used inhalants in the past year. Methemoglobinemia has favorable outcomes with treatment. We present the case of a 43-year-old man, with a medical history of type 2 diabetes mellitus and hypertension presenting with bluish discoloration of the fingers that progressed to his tongue and lips. He disclosed using a recreational inhalant called “Rush” before coming in. Vital signs showed low oxygen saturation on room air, and further investigation revealed elevated methemoglobin levels on the venous blood gas. Methylene blue was used for treatment, with complete resolution of the patient's symptoms and the only side effect of green urine. The patient was later discharged home in a stable condition. History taking is a crucial part of managing methemoglobinemia, as presentation to the clinical setting could vary. Our case presents the milder version of the disease with quick complete recovery after appropriate treatment. It demonstrates inhalant abuse in a less common age group. The case also demonstrates a common side effect of methylene blue that is often forgotten.

## Introduction

Nitrites, commonly called "poppers," were first marketed in small glass ampoules that users would crush to release nitrate vapors into the air, which were then inhaled for medicinal use [1]. The recreational use of nitrites for their euphoric effects can lead to methemoglobinemia, a condition characterized by an abnormal increase in methemoglobin (MetHb) levels in the blood, which reduces the blood's capacity to carry oxygen effectively [2]. According to the Substance Abuse and Mental Health Services Administration (SAMHSA) in 2021, 2.2 million people, or 0.8% of people aged 12 and older, were reported to use inhalants in the past year [3]. Percentages decrease with age, with the highest age group being 12 to 17 years old at 2.4% [3]. No difference in inhalant abuse was seen between ethnic groups. Methemoglobinemia can significantly impair oxygen delivery to tissues and necessitates prompt medical intervention. Presenting symptoms are thought to be on a spectrum and include cyanosis, pallor, fatigue, central nervous system depression or seizures, metabolic acidosis, dysrhythmias, and death [2]. Here, we present a case of a male patient who developed methemoglobinemia following the inhalation of nitrites for recreational purposes.

## Case presentation

A 43-year-old male patient presented to the emergency department with a complaint of bluish discoloration of his fingers, lips, and tongue. The patient had a past medical history of uncontrolled type 2 diabetes and hypertension on no current medication. He did endorse a history of inhalant abuse in the past but no history of intravenous (IV) drug abuse. Prior to the onset of symptoms, he used an amyl nitrate-containing inhalant known as “Rush.” Despite the history of inhalant abuse, the patient denied any previous history of skin discoloration; however, he mentioned this time he used more than he usually does. He noticed a bluish discoloration of his fingers before going to sleep. When he woke up 1 hour later, he noticed additional discoloration of his lips and tongue, prompting his presentation.

Upon arrival at the outside facility, the patient had an oxygen saturation as low as 85% on room air; otherwise, his vitals were within normal limits. A physical examination revealed dusky fingers, with no signs of distress, the cardiac examination was normal, and the respiratory examination revealed clear lungs bilaterally. A 12-lead electrocardiogram (EKG) showed normal sinus rhythm. Laboratory investigations were within normal limits (Table 1).

**Table 1:** Laboratory blood work

Test	Results	Reference range	Units
White blood count	6.6	3.2-11.6	10^3^/uL
Red blood count	4.6	3.99-6.0	10^6^/uL
Hemoglobin	14.1	12.6-17.9	g/dL
Hematocrit	41.7	38.9-50.1	%
Platelets	178	149-451	10^3^/uL
Sodium	140	136-145	mmol/L
Potassium	3.8	3.5-5.1	mmol/L
Chloride	109	98-107	mmol/L
Bicarbonate	23	23-31	mmol/L
Glucose	169	82-115	mg/dL
Creatine	0.6	Not reported	mg/dL
Blood urea nitrogen	14	Not reported	mg/dL
Magnesium	1.9	1.6-2.6	mg/dL
Phosphate	4.2	2.3-4.5	mg/dL
Thyroid-stimulating hormone	0.5	0.46-4.68	uIU/mL
Lactic acid	1.6	0.5-1.9	mmol/L

Urine drug screen (UDS) was negative. Venous blood gas (VBG) showed an elevated MetHb level of 12% (Table 2).

**Table 2:** Venous blood gas

Test	Results	Reference range	Units
pH	7.46	7.38-7.46	-
PCO_2_	35	30-44	mmHg
PO_2_	Not calculated	80-106	mmHg
HCO_3_	25	19-29	Mmol/L
Methemoglobin (MetHb)	12	0-1	%

Chest X-ray did not reveal any acute intra-thoracic process.

The patient was placed on supplemental oxygen via a nasal cannula of 4L. Also, 100 mg of methylene blue IV was administered, and a follow-up VBG showed normalization of MetHb levels. Clinical improvement was also noted, and the patient was weaned off oxygen. Urine analysis was obtained after treatment due to a change in urine color to green (Table 3).

**Table 3:** Urine analysis

Test	Results	Reference range	Units
Color	Green	Yellow	-
Clarity	Clear	Clear	-
Glucose	250	Negative	mg/dL
Bilirubin	Small	Negative	-
Ketone	+3	Negative	-
White blood count	<5	<5	hpf
Red blood count	<5	<5	hpf

The patient was later discharged home in stable condition from the intensive care unit.

## Discussion

The chemical found in “poppers” belongs to a class of drugs called alkyl nitrites. This includes amyl nitrate, butyl nitrite, isobutyl nitrite, isopropyl nitrite, and cyclohexyl nitrite [4]. Recreationally, amyl nitrite products are frequently sold under several brand names on the street: Rush, Super Rush, and Jungle Juice, among others [1]. In the medical field, amyl nitrite functions as a vasodilator, a drug that dilates blood vessels. It lowers blood pressure and increases heart rate. Physicians may prescribe it to alleviate angina, characterized by chest pain, tightness, or squeezing. On the other hand, recreationally, people particularly use it during intercourse. It is particularly popular amongst homosexual men because, in addition to the brief “high” a person receives, it can also help relax the muscles in the anus [1]. Some of the common adverse effects range from mild skin irritation to life-threatening methemoglobinemia.

Methemoglobinemia is associated with oxidization of divalent ferro-iron of hemoglobin (Hb) to ferri-iron of MetHb, which has an impaired ability to irreversibly bind oxygen. Allosteric modifications brought about by iron in the ferric (Fe3+) state allow for the irreversible binding of oxygen. The oxygen-dissociation curve of Hb is shifted to the left by the matching ferroglobins in the tetramer. Due to this change, ferric iron has a higher affinity for oxygen, which impairs oxygen release into the tissue [2]. It can occur either due to a congenital process or due to an acquired process. The congenital forms occur either due to autosomal recessive defects in the enzyme cytochrome b5 reductase (CYB5R) or due to autosomal dominant mutations in the genes that code for globin proteins, known as hemoglobin M [5]. Acquired causes are much more common: exposure to direct oxidizing agents (e.g., benzocaine and prilocaine), indirect oxidation (e.g., nitrates), or metabolic activation (e.g., aniline and dapsone) can result in methemoglobinemia [6].

Under physiologic conditions, the body oxidizes small amounts of ferrous iron to ferric iron (Fe3+) when it routinely delivers oxygen to the tissues. However, the level of MetHb is usually below 1% with the help of the enzyme cytochrome-b5 reductase [7]. Cytochrome-b5 reductase uses nicotinamide adenine dinucleotide (NADH) formed during the glycolysis process to perform its function [7]. Methemoglobinemia due to toxic exposures, such as this case of using “poppers,” occurs when cytochrome-b5 reductase’s ability to reduce MetHb is impaired due to the overwhelming oxidation stress causing an increasing percentage of MetHb in the body [7].

A wide range of presentations were seen in different methemoglobinemia cases. Lefevre et al. describe a case report of methemoglobinemia caused by "poppers" [8]. Although treatment with high-flow nasal oxygen and methylene blue was similar to that in our case, they described very different features. The patient presented in a coma state after ingesting a combination of ethanol and “poppers.” The patient was in respiratory distress and had extensive gray skin discoloration. Also, the patient had hemoglobin levels that were six times higher and lactate levels that were four times higher compared to our case. In the reported case, the venous blood analysis showed a chocolate-brown color, indicative of severe methemoglobinemia. Another case of methemoglobinemia induced by amyl nitrite "poppers" inhalation was reported by Barry and McAteer [9]. Although their case shares a common etiological agent with our case, it exhibited different characteristics. The patient presented with a syncopal episode, blue nail beds, and perioral cyanosis following the ingestion of three bottles of “poppers.” The trigger of his syncope was speculated to be a hot shower after the ingestion of the poppers. Barry and McAteer explain that the syncope could have been vasovagal in nature, resulting from the combined vasodilatory effects of the popper and hot shower [9]. Gooley et al. also describe a case of ventricular fibrillation after the ingestion of an isobutyl nitrite "popper" product [10]. The patient did not have methemoglobinemia. However, he was found in cardiac arrest after the inhalation of a bottle of acetone-free nail polish containing isobutyl nitrite, indicating more severe complications of inhaled nitrites.

A MetHb assay along with blood gas analysis were the initial diagnostic steps in most reviewed cases. Sonck et al. report a case of a 38-year-old man who presented with cyanosis, dyspnea, and hypoxia that did not improve with oxygenation [11]. The patient did not reveal the use of any substances upon initial questioning. An arterial blood gas (ABG) analysis was ordered, revealing acidosis, elevated lactate, and normal oxygen saturation. MetHb level was obtained, which turned out to be elevated, and the diagnosis of methemoglobinemia was proposed [11]. Similar to our case, Barry and McAteer [9] used a venous gas analysis and MetHb levels to confirm the diagnosis of methemoglobinemia. Elgendy et al. added that the diagnosis of methemoglobinemia can be suspected by a suggestive history of the use of “poppers” and confirmed by ABG analysis, utilizing a co-oximetry to directly measure the oxygen saturation rather than calculating it from the partial pressure of oxygen, which is usually inaccurate [1]. An additional physical finding that can help confirm the diagnosis of methemoglobinemia is the presence of chocolate-covered blood, which was noted in the cases by Lefevre et al. [8] and Reisinger et al. [12].

Methylene blue has been proposed for the management and treatment of methemoglobinemia. It reduces the oxidized form of hemoglobin Fe3+ to Fe2+, which increases the oxygen-binding capacity of hemoglobin and the delivery of oxygen to the tissues [13]. In the case reported by Tello et al., treatment consisted of a methylene blue infusion at 1 mg/kg over a 20-minute duration in addition to supplemental oxygen [14]. Similarly, Ribeiro Paixão et al. describe the need of using repeated doses of methylene blue to achieve normalization of the MetHB levels [15]. Although rare, pediatric popper-induced methemoglobinemia has been reported. Toutain et al. described the case of a 3-month-old boy who accidentally ingested 5 mL of “poppers” after the father had mistaken them for the vitamin D container [16]. Similar to our case, the patient’s symptoms rapidly improved following methylene blue (1 mg/kg over 60 minutes). Notable differences in the patient’s initial presentation were the delayed capillary refill and the lack of cyanosis.

In the case reported by Reisinger et al., 240 mg (3 mg/kg) of toluidine blue was used for treatment instead of methylene blue due to its slightly enhanced efficacy and fewer side effects [12]. In their case, the patient presented with MetHb levels that were four times higher compared to our case. In some cases, management of methemoglobinemia can be supportive. In Barry and McAteer’s case [9], the patient did not require methylene blue even though the MetHb levels were twice as high as in our case. Monitoring and IV fluids were sufficient to resolve the patient’s symptoms. The vast majority of cases do respond well to treatment without any sequel [2].

The green urine discoloration noted after administration of methylene blue is a known side effect [17]. It follows a dose-dependent pattern and is self-limited. Interestingly, Koratala and Leghrouz utilized this feature of methylene blue to assess the integrity of the gastric wall following an exploratory laparotomy [18].

Hwang et al. describe a case of a 28-year-old man who used sodium nitrite (a commercial coloring agent, and food preservative) to commit suicide [19]. The characteristic dark brown face and bright red oral mucosa were described at the time of the autopsy [19]. Additionally, they noted that MetHB levels up to 20% are well tolerated, levels above 40% cause cardiopulmonary symptoms, and levels at 70% are lethal [19]. However, Cvetković et al. describe a case of sodium nitrite poisoning resulting in the death of a 70-year-old man with a MetHB level of 9.8% [20]. Our patient showed cardiopulmonary symptoms with hypoxia at the time of presentation with a MetHB level of 12%.

## Conclusions

Methemoglobinemia is a life-threatening condition that can result from nitrite abuse. Although common in younger age groups, our case demonstrated that inhalant abuse should also be considered in older patients. Clinical presentation can vary based on severity. Our patient exhibited milder symptoms, but more severe symptoms were reported. Diagnosis can be reached clinically and confirmed by a blood gas showing elevated levels of methemoglobin, as was seen in our case. The most commonly utilized treatment is methylene blue. As with our patient, complete resolution is achieved with prompt and early treatment. Methylene blue can result in green urine, which was shown in our case, and this is an often forgotten side effect. Patient education and awareness are important to avoid recurrences, especially in a patient like ours with a history of inhalant abuse.
